# COVID-19 Daily Realities for Families: A South African Sample

**DOI:** 10.3390/ijerph19010221

**Published:** 2021-12-25

**Authors:** Kezia Ruth October, Lisa Rene’ Petersen, Babatope Adebiyi, Edna Rich, Nicolette Vanessa Roman

**Affiliations:** Centre for Interdisciplinary Studies of Children, Families, and Society, Faculty of Community and Health Sciences, University of the Western Cape, Cape Town 7535, South Africa; 3177256@myuwc.ac.za (L.R.P.); atommega@yahoo.com (B.A.); erich@uwc.ac.za (E.R.); nroman@uwc.ac.za (N.V.R.)

**Keywords:** family, family experiences, COVID-19, pandemic, South Africa

## Abstract

The COVID-19 pandemic affected families globally. Empirical research has been explored to understand the impact of COVID-19 on families across countries, however, there are limited findings of how COVID-19 has affected the daily realities of families in South Africa. This study used an exploratory qualitative research approach to explore the experiences of COVID-19 for South African families. Findings suggest that the negative outcomes of COVID-19 experienced by South African families included a shift in the daily routines, restrictions on family events, lack of socialization and loss of connections, family conflicts, financial constraints as well as psychological impacts. On the contrary, the positive outcomes included increased family time and communication, cleanliness, and good health status, and improved financial management. Implications for future research should include research focused on the health impacts of COVID-19 on diverse family structures, family compositions, and family dynamics. In-depth research and findings can assist in developing policies and interventions for families.

## 1. Introduction

The COVID-19 pandemic has affected the lives of many globally. In December 2019, the Corona Virus was initially originated in Wuhan, China. The virus known as COVID-19 has since spread globally affecting more than 229 million people, with death rates of over 4.70 million [1]. On 11 March 2020, the World Health Organization (WHO) has since declared COVID-19 a global pandemic. By September 2021, the global infection rate was at 230,326,827 cases, and 4,722,924 deaths were recorded [1]. The COVID-19 virus has been found to generally spread between individuals through their respiratory droplets from coughs and sneezes [2,3]. The symptoms of the COVID-19 virus have been found to be similar to that of influenza including shortness of breath, severe coughs over time, and a fever. Findings suggest that after exposure to the virus, individuals can be carriers of the virus from 2 days to 2 weeks [4]. At the onset of the pandemic, organizations such as the WHO, coupled with governments worldwide, worked together to put contingency plans in place to prevent, combat and support the health care systems, which were under strain as a result of COVID-19. Some of these contingency plans set in place included testing by means of nasal and throat swabs, screening at numerous venues, social distancing, and lockdown only to mention a few. This pandemic and the implemented contingency plans have influenced the lives of many families in numerous ways.

One of the main contingency plans set in place by governments globally was lockdown restrictions. In the South African context, these restrictions were introduced in a risk-based, five-level approach. The approach was guided by numerous factors, which include but are not limited to, the transmission and infection rate of COVID-19, as well as the capacity of the healthcare system to accommodate patients [5]. In descending order, the 5 alert levels started with lockdown level 5—which indicated high COVID-19 transmissions and low healthcare readiness—and with time eased down to level 1—indicating low transmissions and high healthcare readiness [5]. At the start of the pandemic, the transmission and infection rates in many South African communities were increasing rapidly. Therefore, the initial lockdown restrictions were implemented to flatten the curve of infection and slow the spread of COVID-19. Level 5 lockdown was first implemented on 26 March 2020 [6]. Consequently, healthcare workers had the chance to prepare and equip themselves with the necessary precaution measures. The level 5 lockdown also allowed the public health sector to bolster testing and prevention interventions, and with time, the transmission and infection rate decreased. The lockdown levels then eased from level 5 until they reached level 1 in September 2020. However, the country experienced a resurgence of COVID-19 cases, which resulted in an adjusted alert level 3 being implemented [5]. The trend of infection and transmission then decreased, and the country was placed back on alert level 1 between March and May of 2021. However, a new variant-the Delta variant-spread rapidly throughout the country, moving the lockdown restrictions from level 1 to level 2 in May, and then to level 4 in June 2021 [7]. Thereafter, the country moved between levels 3 and 4, and by October 2021, back to level 1.

Moreover, the aforementioned lockdown regulations included restrictions of interaction and mobility of the population, curfews, travel restrictions-both national and international-, business activity restrictions, closure of schools and universities, as well as the cancellation of events and gatherings [8,9]. As a result, COVID-19 and its associated lockdown restrictions, impacted families in different aspects of life. Therefore, this paper discusses the COVID-19 daily realities of South African families.

### 1.1. Families in South Africa

The family plays a pivotal role in the promotion of good societal outcomes. As such, the family aids in sustainable development across all levels of society. As a functional system, the family largely contributes towards both economic and social development, including the promotion of solidarity and distribution of resources in households and broader societies [10]. While the family is defined by two or more individuals related by blood, marriage, or adoption and who live together [11], nuclear families are no longer regarded as the typical family form within South African households [12]. In South Africa, diverse family structures, such as single-parent households, skip generation households, and child-headed households amongst others, significantly influence the process of family functioning [12]. Well-functioning families further facilitate and promote good family outcomes and family well-being. This is evident in the way a family operates such as providing members a foundation that includes: social identity, economic support, nurturance, socialization, protection, and care that ensures successful development and adaptation to function and flourish within broader society [12]. However, although well-functioning families contribute to positive family outcomes, the absence of an enabling and supportive environment significantly impacts family well-being and functioning [13]. For example, stressful life events, such as adverse socio-economic challenges and health conditions, can negatively affect the family processes and functioning, leading to family dysfunction which affects the family’s overall well-being [14]. The Department of Social Development [12] further argues that COVID-19 played a significant role in consequently impacting the well-being of families in South Africa. This is evident in the changes within the burden of care, health concerns, and the loss of family members.

### 1.2. Impact of COVID-19 on Families

The outbreak of the pandemic had more than just morbidity and mortality implications for families. It also led to mandatory COVID-19 containment measures which include lockdown, quarantine, and social distancing. Consequently, this had both positive and negative implications for families. For many, family life has been uprooted which meant changes to their ‘normal’ everyday routine. The lockdown regulations resulted in many families now working from home and homeschooling. With schooling facilities (i.e., includes daycare and tertiary institutions) and many offices being closed, there has been a shift in responsibilities and roles, particularly for parents. For example, parental roles now include childcare, academic teaching [15], all while working from home.

This meant that families, particularly those within the same household, would be spending more time together. Consequently, communication in the family has been impacted as lockdown provided opportunities for families to share and talk about their emotions and feelings [16]. As parents and children were spending more time together, this created opportunities to build stronger relationships amongst family members. While this is true for some families, others experienced family feuds and conflict in their homes, as spending more time together resulted in frustration and arguments. For example, families spending more time at home also meant more women (and children) were at risk of being victims of violence in the home [17].

Additionally, the lockdown and social distancing regulations meant that direct interpersonal interactions were prohibited, which resulted in fewer opportunities to visit extended family, friends, and neighbors [18]. Therefore, the social isolation brought about by lockdown restrictions, had psychological impacts for many. Being connected to others can act as a coping mechanism, particularly during times of distress, thus being isolated from family and friends often leads to feelings of distress and loneliness [19,20].

The well-being of family and friends combined with work stressors are challenging many families face during the ongoing COVID-19 pandemic [21]. Waite and Creswell [21], found that in some families, parents are unable to meet the needs of workers as well as the needs of their children; thus, adding more stress. In South Africa for example, many families come from impoverished neighborhoods, and with the outbreak of the pandemic, many people lost their jobs [8]. Research evidence further suggests that there has been an increase in the unemployment rate and economic uncertainty has been reported [22]. This in turn led to financial stressors. For example, with schooling moving online, many families and children were at a disadvantage as they did not have the required resources or finances [23]. As a result, contingency plans, such as the Temporary Employee Relief Scheme (TERS), were introduced to ease the effects of the pandemic and job losses. However, this did not benefit or help all citizens in need of the TERS, as some citizens did not meet the criteria, or the employers did not submit the required documentation [24].

Moreover, the onset of COVID-19 also increased the awareness of health and cleanliness. As a means to assist in preventing or reducing the spread of the virus in the South African government implemented health and safety protocols. For example, the protocols set in place include mandatory wearing of masks in all public spaces, the washing of hands, and sanitizers being provided at all institutions including malls and offices [25]. These protocols were implemented as the COVID-19 virus is spread mainly through direct and indirect (on surfaces), or close contact with infected persons. Therefore, the washing of hands and covering the nose and mouth is of utmost importance for the prevention and spreading of the virus [26]. These regulations have resulted in behavioral hygiene changes as families now incorporate the washing of hands, sanitizing, and the wearing of masks, whenever they leave the home.

Although studies have been explored to understand the impact of COVID-19 on families across countries, there remain limited findings of how COVID-19 has affected the daily livelihoods of families in South Africa. Further in-depth understanding of the impact factors of the pandemic outcomes is significant in order to both address and develop resources and interventions for families during the pandemic [27].

### 1.3. Study Aim and Objective

The study, therefore, aimed to explore the COVID-19 daily life of South African families which was guided by the research question: What is the everyday life of families during the COVID-19 pandemic in South Africa?

## 2. Materials and Methods

### 2.1. Study Design

The exploratory qualitative research design was used to explore the perspectives of South African families’ daily life of COVID-19. This research design was preferred as empirical research on COVID-19 is limited globally. The exploratory research design was used to gain a deeper understanding of the daily experiences of COVID-19 by South African families [28]. This method of research inquiry will provide initial information about the phenomenon under study-South African families and the COVID-19 pandemic daily realities.

### 2.2. Sampling Procedure

The snowball sampling method was utilized to recruit participants due to COVID-19 lockdown. Participants were drawn from each of the six municipal districts of the Western Cape Province. Participants were selected using the following criteria: (1) If they were eighteen years and older; (2) If they were members of a South African family; (3) If they can speak and understand English, Afrikaans, or isiXhosa; and (4) If they lived in Western Cape Province. The recruitment of participants was advertised on various websites (University websites and other organizations) and other social media platforms (WhatsApp groups). All the participants who met the criteria were selected and they were asked to assist in the recruitments of other participants who also met the criteria. The total number of participants included in the study was *n* = 31 which was made up of *n* = 13 males and *n* = 18 females (See Table 1). The average age range of participants was between 25–35 years of age. Majority of the participants identified as living within a nuclear family structure. Lastly, more participants were employed (67%) than unemployed (32%) in the study.

### 2.3. Ethical Considerations

Permission for the research was granted by the Senate Research and Ethics Committee at the University of the Western Cape (HS20/5/4). The information sheets that contained the aim and roles of participants were sent to the participants. After reading, those participants who were willing to participate were asked to sign consent forms. Thus, informed consent was obtained from all subjects involved in the study. The participants were informed that participation is voluntary, and they can withdraw from the study at any time, without penalty. The research team saved all the information obtained from participants on a computer, protected by a password, to ensure confidentiality. No participant names were used during data analysis to ensure anonymity.

### 2.4. Data Collection

Taking into consideration the COVID-19 restrictions, semi-structured interviews were conducted in English, Afrikaans, or isiXhosa; through telephonic conversations and were audio-recorded with participants’ consent. The interviews were conducted by the members of the research team using an interview schedule (see Table 2). The interviews lasted for an average of 40 min and were conducted between June and September 2020. Participants were asked about their COVID-19 daily realities. After conducting each interview, the interviewer constantly went over the recordings to ascertain if the data collected is saturated. Data saturation was reached when no ‘new’ information was elicited from the participants and subsequent participants recruited, had similar demographic variables, with the previous participants [29]. Therefore, the interviewer canceled the subsequent appointments made to interview other participants [29].

### 2.5. Data Analysis

Data were analyzed inductively using thematic analysis which allows for flexibility in interpreting the data [30]. All the interviews conducted were transcribed. The interviews conducted in Afrikaans and isiXhosa were translated into English. Following transcription and translation, members of the research team read and reread the transcripted data for familiarization and derivation of meanings, and to produce initial codes. The purpose of coding was to reduce the raw data into parts that are relevant to the research question, break down the data into manageable sections, and transform the data. The initial codes generated were reorganized to obtain refined codes. Codes with similar concepts were grouped to form sub-themes and those sub-themes with similar ideas were further clustered to form the final themes. All themes were checked against transcripts for appropriateness. Finally, the themes were described and supported by extracts from transcripts. In addition, data were further analyzed through the ATLAS Ti software program (ATLAS.ti Scientific Software Development GmbH, Berlin, Germany), to identify and categorize topics [31].

### 2.6. Trustworthiness and Rigour of the Study

The credibility, transferability, dependability, conformability, and a reflexive approach to the inquiry and analysis, were used to establish the rigor and trustworthiness in this study [32]. A detailed methodology-the study’s site, participants, and procedures used to collect data-was provided to ensure transferability in this study. The methods of data collection and analysis were described in detail to ensure dependability for the study. Furthermore, a single interview schedule developed by all members of the research team was used for all interviews. The data coding was done by more than one member of the research team. The credibility of the study was ensured by conducting member checking at the end of each interview-a recap of the main points from the interviews was conducted. In addition, participants were allowed to express themselves freely during the interview. To ensure the confirmability of the study, verbatim transcripts of the participants’ responses were provided. A reflective journal is a document that contains the discussions, deliberations, and decisions made by the research team when conducting research. The research team kept a reflective journal as a part of the audit trail when conducting this study.

## 3. Results

The current study aimed to explore the daily life of families during the COVID-19 pandemic. The key findings identified, formed part of a larger study that focused on family life during COVID-19. One of the main themes that emerged from the data analysis included family experiences of COVID-19. The main theme was further divided into six sub-themes (see Table 3) namely: (1) Lockdown restrictions on family routines and events, (2) Family time, (3) Family communication and socialization, (4) Financial management and constraints, (5) Cleanliness and good health and lastly (6) Psychological impact.

### 3.1. Family Experiences of COVID-19

Participants in the study expressed various changes in their daily lives due to COVID-19 and the lockdown regulations. These experiences were further categorized into six sub-themes which were made up of participants’ positive and negative experiences associated with COVID-19 and the lockdown regulations. (1) ‘Lockdown restrictions on family routines and events’ include aspects of COVID that shifted families daily lifestyles; (2) ‘Family time’ relates to aspects of how families connected with each other; (3) ‘Family communication and socialization’ refers to aspects of families’ utilization of social media as well as families experiences of restrictions on social gatherings; (4) ‘Financial management and constraints’ encompassed how families made use of ways to save money and the challenges they experienced financially; (5) ‘Cleanliness and good health’ notes how family experiences of COVID has improved their hygiene and overall well-being; and lastly (6) Psychological impact comprises of factors relating to families mental well-being during the COVID-19 pandemic.

#### 3.1.1. Lockdown Restrictions on Family Routines and Events

With lockdown restrictions being implemented, participants experienced a shift in their family routines and events. Participants indicated that as a result of lockdown restrictions, there have been changes to their daily lifestyles and cancellations of specific events. In addition, participants mostly expressed having the freedom to go out due to the lockdown restrictions. These were expressed as follows:

“*I don’t know what else to, of course not being able to go out and exercise or see friends is a negative… like myself included, but most people, your routine just got completely destroyed*”.

(Male, 28 (age), Software Engineer)

“*Uhm, I miss going to the beach, I miss going away on short breaks. … Uhm, and I miss the freedom that we used to have of just getting in the car and driving*”.

(Female, 54, Retired)

Lockdown restrictions also resulted in changes to work and school routines. At the start of the pandemic, most parents started working from home and children did not attend school in person. One participant highlighted how difficult it was for their children to no longer attend school, while another highlighted that it brings families together.

“*The most difficult aspect has been for my children to stay away from school*”.

(Male, 30, Social Worker)

“*That’s a very good thing. It brings families together also although parents aren’t able to go to the office they are able to spend more time with their children*”.

(Female, 28, Research Assistant)

Working from home was noted to be another change in families’ daily routines. The following participant expressed that working from home meant that they had a lot less stress. This was voiced as follows:

“*Being home together because everybody is working from home. A lot less stressed because not having to commute to the office and then just also with the clients being able to have a lot more meaningful interaction with the entire online sessions because they feel more comfortable and they actually show you the home environment*”.

(Female, 28, Counsellor)

These shifts in normal routines have underlined how the family’s external environment has been impacted by COVID-19. COVID-19 and the lockdown restrictions also influenced family gatherings. Participants expressed that they were unable to celebrate special occasions and had to cancel their plans in light of the pandemic. For example:

“*For myself and my wife it’s been very difficult because a lot of what we had to do in these past months revolved around government documentation which took an extremely long time due to the virus. As well as the fact we weren’t able to get married like we had planned, well we weren’t able to have a full wedding. We only got legally married*”.

(Male, 26, Unemployed)

Taking into account how COVID-19 has impacted family routines and events, one participant voiced the impact lockdown had on their family. The participant indicated that they were not allowed to visit a family member in the hospital and could not say goodbye. This was noted as follows:

“*The negative was the frustration that we could not go home to make sure that our other family members were okay because of the lockdown and the permit. You needed a permit in order to go to Cape Town although it is in the same province. But then you could only get a permit if there was a death in the family. They do not make provision if a family member was sick and family members were old. Like I said my mom is seventy. Yes, she is still in the household with my second oldest brother but for me, it is not the same. I actually wanted to see if she was okay*”.

(Female, 33, Counsellor)

In addition to lockdown restrictions on family routines and events, experiences of family time during COVID were another sub-theme that was found in the data analysis.

#### 3.1.2. Family Time

Participants indicated that due to lockdown and restrictions, as a family they could spend more time together. Common views further expressed that staying at home as well as working from home offered families to become closer to one another through means of eating or supporting each other. These were expressed through the prolonged periods of sharing common spaces as a family. Some participants voiced that this has strained their family relationships.

“*And also just the lockdown probably gave other families time that they never had for the longest of time just to sit together as a family and you know, just maybe have a meal together. I think some families might have found each other… And you know just its quite good that you know we are just together and even during this time, that’s when probably one needs family, or those that he loves the most, to be close to them*”.

(Male, 31, Educator)

In relation to families’ experiences of increased family time, one participant expressed that spending time together as a family allowed them to support each other during the COVID-19 pandemic.

“*Being able to just stay at my house and spend time with myself and my family was a massive benefit… Ja, so, time spent together, number one. Coming together to support one another as well and also from my side just getting a break from the world*”.

(Male, 28, Software Engineer)

While one participant associated spending more time at home with being able to see their children grow, another participant further voiced increased family time allows them to forge better relationships with family members, which were not present before.

“*I got to spend more time with my family, which is always positive. I got to see my children grow substantially how long has it been in the five months that I’ve been home. My eldest son is starting to write*”.

(Female, 28, Administrative Clerk)

“*We built a closer relationship with my sister’s kids, which was non-existent before*”.

(Male, 28, Self-employed)

Contrastingly, participants also expressed that increased family time has led to an increase in family feuds, which has strained family relationships.

“*We got on each other’s nerves because of constantly being around each other*”.

(Female, 26, Law student)

“*I think that we need to be aware of the fact that some families are strained having spent so much time indoors with each other that the cabin fever is starting to affect their relationships*”.

(Male, 27, Counsellor)

#### 3.1.3. Family Communication and Socialisation

Overall, participants expressed that there was increased use of social media platforms such as group chats, to communicate with each other and stay in touch. COVID-19 has had a positive effect on families as families are staying more in touch and communicating with each other, now more than they have before. Contrastingly, participants also expressed their frustrations regarding how lockdown has impacted their relationships with family. These were expressed in the loss of communication and lack of socialization amongst family.

The following quotes express participants’ increased communication using social media.

“*Okay…we using technology better to keep in touch. So, we do like, keep in touch more regularly, whereas, in the past, we might go for a week or two not really like checking in on people. So that is a good thing… So, I think you kind of learn to be better with people. Whereas in the past, you can just like go out if you like if this is too much for you or whatever, and just avoid like talking about issues and stuff. So, and then in that way, maybe everyone is become more caring and understanding*”.

(Female, 35, Attorney)

“*So at the beginning obviously when it was complete national lockdown, so it was obviously tough. You could not meet them anywhere. The only means was to sort of communicate telephonically, virtually, calling and things like that. So that was the only way that you could have any type of interaction and of course that eased up with the restrictions being able to go out*”.

(Female, 41, Private Banker)

“*And I think that communication has improved. Within my family and speaking between myself and Eugene, but then I am also speaking about the broader family like for example, this WhatsApp group, we now have, we did not have before*”.

(Female, 46, Monitoring and Evaluation officer)

Conversely, participants also expressed their lack of communication and socialization amongst family members. Some participants voiced feelings of longing towards their families. This was noted as follows:

“*a lack of communication in general, like, because you want to socialize and you can’t*”.

(Female, 28, Nail technician and beauty therapist)

“*The fact that nobody could see my grandfather for about two, three months because the old age home locked down completely and you weren’t allowed in and out*”.

(Female, 28, Counsellor)

One participant noted the social impact of working from home rather than from the office and the difference in the level of personal interaction between others. This was expressed as follows:

“*And the way, the way that it affects people working from home. They feel isolated quite often. I interact quite on a daily base with the team that I work with. So we have Zoom calls and we have team meetings and things like that. So I’m quite self-sufficient and I’m quite happy with it. But I do miss the interaction because we use to, uhm, everyday meet with a group of people for lunch, sit together and chat, so you do miss that personal interaction. And it’s not the same doing it remotely*”.

(Female, 64, Administrator)

Another sub-theme that was found in the data analysis included financial management and constraints.

#### 3.1.4. Financial Management and Constraints

COVID-19 led to changes in family lifestyles which influenced how families manage their finances. Factors in relation to financial management were expressed by the following participants. One participant expressed that telecommuting has assisted in saving money spent for traveling to work. This was voiced as follows:

“*I would say that before that, I had some financial battles I’m not going to lie but because I was not retrenched or anything my work continues as I was working from home and it actually made me save money now as an individual… To save money, not on spending money, transportation and stuff so at least I can do something better after this whole thing*”.

(Female, 26, Graduate Intern)

Another participant noted an improvement in working with their finances:

“*Family has learned how to better use their money, I feel*”.

(Male, 26, Unemployed)

On the contrary, two participants highlighted their concerns regarding financial effects and financial uncertainty they experienced due to COVID-19 and lockdown regulations. These included socio-economic factors such as unemployment and income loss. These were noted as follows:

“*I think one of the biggest concerns that I been thinking about a lot is with the COVID and especially during the level five lockdown, many people were for so long out of work that it made it even impossible for them to come back to a working environment and the consequences of that has not really been saw through or considered in a particular family or household*”.

(Female, 33, Counsellor)

“*I suppose the negative was my husband could not work it affected him, he could not operate so that was the other issues. I think financial it was a strain in the sense of not being productive. His staff was affected because obviously there is no work to do, they cannot get paid. So that was a bit of a hard one to deal with in that sense. But of course being locked down and obviously not knowing bad things were going and spiralling because of the virus but at least we were indoors and not interacting with many people or anything like that*”.

(Female, 41, Private Banker)

Another sub-theme that emerged from the data analysis included the act of Cleanliness and good health.

#### 3.1.5. Cleanliness and Good Health

The COVID-19 pandemic also introduced health and safety protocols, which enhanced the awareness of hygiene. Participants voiced that due to COVID-19 a positive outcome was taking better care of their health and practicing cleanliness. With regard to health, participants expressed a change in their eating behavior as participants expressed that they developed a stronger awareness of what they eat. Consequently, participants noted a change in their diet which now includes eating healthier and cooking food at home as opposed to going out to eat. This was voiced as follows:

“*I think health-wise we became a little bit more healthy because most of the places was shut down and we predominantly started making our own food and we became more aware of what we are putting in and also we became a little bit more active*”.

(Female, 33, Counsellor)

In addition to eating healthier, participants expressed a decrease in occurrences of getting sick due to the better practice of cleanliness. Participants viewed this as a positive factor and reinforcement of evidence of better hygiene practice. This is evident by the following participant statements:

“*like another positive thing about this thing my kids weren’t sick… I don’t think they were at the doctors once this year. And usually by this time my daughter, because she has got high allergies, so when she is in school this time, she is already struggling with her chest and stuff, and she is quite healthy. So that is another positive thing about this whole thing that, even though the kids couldn’t go to school, they weren’t sick at all*”.

(Female, 28, Nail technician and Beauty therapist)

“*I’ve hadn’t had colds this year. Sanitizing obviously been good to us. Because we pretty much make a lot of Cold and Flu meds and I think it’s cold and flu season hasn’t been as intense as we would have expected it. And I’m assuming a lot of that is probably down to cleanliness and, and social distancing*”.

(Female, 56, Tech business).

While cleanliness showed how COVID-19 greatly contributed to families’ health and well-being, negative experience of COVID-19 was noted on families’ psychological health and well-being.

#### 3.1.6. Psychological Impact

Most of the participants expressed feelings of stress, fear, depression, and anxiousness surrounding both COVID-19 as well as lockdown. These feelings have impacted both their day-to-day routines, through lockdown restrictions, and their overall psychological health.

“*It’s not nice because you feel lonely and basically you get, you get, some, some of them, like you can get depressed*”.

(Male, 19, Student)

One participant expressed that newfound anxiety was related to the fear of being judged most of the time as well as the fear of ending up in the hospital. This has highlighted the issue relating to the stigma effect of COVID-19. This is driven by the fear of the unknown. On the other hand, another participant expressed the association between the fear of the COVID-19 virus and the effects it can have on people.

“*A deeper form of anxiety is created by being judged all the time*”.

(Male, 28, Self-employed)

“*I think the fear and the stress that it put on people. You know, you just don’t know when it’s going to hit you, you don’t know how it’s going to react, you don’t know who’s got it and who hasn’t they don’t even know. I don’t even know if I’ve got or I haven’t so I guess the learning is sort of isolation the fear and you know*”.

(Female, 56, Tech Business Partner)

“*I think a lot of people just didn’t make it in this time and I think it is because of the added stress and not just to mention like, like I know a lot of people that like fell pregnant in this time with me and a lot of them miscarried*”.

(Female, 28, Nail technician and Beauty Therapist)

From the aforementioned statements, it is apparent that participants experienced adverse psychological impacts due to COVID-19. It can be noted that factors such as stress, fear, depression, and anxiety, impacted the daily routines of participants. These findings suggest that participants linked their anxiety with the social stigma associated with COVID-19, as they fear being judged. These negative stereotypes can further prompt the social isolation of groups. Another key concern that also emerged concerns, pregnant mothers having miscarriages.

## 4. Discussion

The study explored the daily realities experienced by South African families during the COVID-19 pandemic. The findings of the current study suggest that the lockdown restrictions imposed by government regulations, to combat COVID-19, have impacted families’ daily lives. Findings further highlighted that COVID-19 and the lockdown regulations had advantageous and disadvantageous for South African families, in relation to their shift in daily routines and cancellations of family events. Similarly, the findings suggest that while families expressed an increase in time spent with family members, this has also led to an increase in family conflicts. Furthermore, while family communication amongst families improved using social media, families had also found they had lost family connections through their lack of physical socialization. The findings on family financial management and constraints have shown that while families have been able to learn how to save on their expenditures, they have consequently also experienced a loss in financial stability due to job loss and unemployment. The study’s results also suggest that the government regulations introduced health and safety protocols, which resulted in families practicing cleanliness more often, and being more health-conscious; and lastly, COVID-19 had immense psychological impacts on families. The psychological impact COVID-19 had on families has emphasized how the lockdown regulations such as social distancing, self-isolation, quarantining, and restriction of travel; affected the mental health of families in South Africa. Although some of the safety measures of COVID-19, such as hand sanitizing, have had a positive impact on families’ health and well-being. The findings of the current study, however, show that there are interconnected factors in relation to COVID-19 and lockdown regulations, that can have both favorable and unfavorable effects on the families’ daily realities.

### 4.1. Lockdown Restrictions on Family Routines and Events

Research studies suggest that the COVID-19 pandemic imposed a vast majority of changes within families’ daily lives. the current pandemic uniquely affected children and families by disrupting daily routines, changing the dynamics of family relationships and roles; and altering the usual childcare, school, and recreational activities [33]. The research found that the lockdown restrictions and regulations have impacted families’ physical activity across all age categories [34]. Globally, the lockdown regulations have restricted access to recreational facilities such as sports, exercise, picnic, and playground environments. The mandatory lockdown regulations that have been imposed in South Africa, as a means to aid in the decrease of COVID-19 infection rates, show that the safety measures enforced were successful in increasing social distancing and thus, overall infection rates [35,36]. Conversely, the current study’s findings show that lockdown restrictions have greatly affected families’ daily routines and family events, changing the way families function both internally and externally to their family environment. This was noted when families were unable to freely visit family members, and special events such as weddings were canceled, which, in turn, affects the family social environment. The restrictions, however, consequently influenced the mobility of families through the exemption of traditional activities such as restricting religious and social gatherings, as well as restricting outpatient and elective visits at health facilities, resulting in the reduction of social contact with extended family and friends [37]. Additionally, the regulations regarding the limited number of attendees at funerals made the loss of a loved one, even more unbearable [38]. Research findings in the UK on lockdown restrictions have shown that these restrictions have had implications on the physical activity of children [39]. Spinelli et al. [40] contrastingly argued that the lockdown has led to an increase in parental stress as parents were responsible for balancing a multitude of factors relative to their duties such as work, child education, and personal events; without any assistance from others. They had to take on the role of being the mother, teacher, friend, entertainer, and caretaker. Similar results were found in a study conducted in Singapore in which lockdown negatively influenced family roles such as poorer work-family balance and increased parental stress and marital conflicts [41]. Roos and colleagues [17] further argue that these factors are associated with lowered levels of parenting quality and impaired child health and development outcomes. In addition, studies conducted in South Africa further indicated that prolonged patterns of isolation and social distancing, have consequently predisposed feelings of loneliness [42,43].

### 4.2. Psychological Impact

Similarly, Ansari and Ahmadi Yousefabad [44] noted that changes in family daily activities can potentially increase family anxiety and stress through prolonged quarantine, resulting in the deterioration of the family’s mental health. The study’s results on the psychological impact of COVID-19 on families stressed how changes in the environment and social spheres, can adversely affect families’ emotional and mental well-being; further underlining the social stigma attached to COVID-19. Global research evidence found that social stigma associated with COVID-19 have contributed to the increased mental distress which includes stress, anxiety, and depression amongst healthcare professionals, individuals who have traveled abroad, individuals who are from specific ethnic groups such as Asian descent, as well as individuals who have already experienced COVID-19 [45]. Studies on the social stigma (e.g., discrimination and devaluation by others) associated with COVID-19 [46] have found that these negative stereotypes can be linked to individuals’ lack of knowledge and information about COVID-19, such as how it can be transmitted, treated, and prevented [47].

### 4.3. Family Time

While the findings on the psychological impact of COVID-19 experienced by families show that these can have adverse effects on the mental health and well-being of South Africans. Studies relative to COVID-19 have also discovered factors of family conflict and its connection with unfavorable outcomes among children and adolescents [48]. The findings of the current study found that increased family time can potentially contribute to family conflicts. Empirical evidence suggests that family conflict and cohesion, are vital influential factors of physical and mental health outcomes, in which families have been exposed to adverse conditions such as financial strains [49,50]. While increased levels of conflictive interactions, within family environments, may contribute to family dysfunctions and disconnections; contrastingly family environments promoting closeness and connected interactions-with family members and extended family members- can mitigate psychological stressors [48]. The findings of the current study show that lockdown has also contributed to an increase in spending quality time with family members and prioritizing family relationships, which promoted family togetherness and social support, amongst families. Toran et al. [51] studied the quarantine process of the experiences of Chinese and Turkish parents. While both negative and positive experiences were noted in the study, Toran et al. [51] underlined that the home quarantine process has changed the daily lives of parents and children. The findings relative to the positive experiences highlighted that both Chinese and Turkish parents were able to improve their family relationships. Through the home quarantine process, both Chinese and Turkish parents were able to spend more time with their children at home and interact with them. Conversely, a variety of family research has found that family connectedness is a protective factor that can buffer challenging outcomes related to stressful conditions [52]. The current study’s findings on family time highlight how COVID-19 contributed to the formation of closer social bonds amongst families, that were non-existent and increased overall appreciation of each other during. Similarly, these findings are supported by another study conducted by Zeng and colleagues [52], which found that family cohesion mitigated the fear of COVID-19 resulting in positive parent-child outcomes, in stressful conditions. Additionally, emerging research on COVID-19 has also found a change in societal norms and traditional gender roles for men and women. While some studies suggest that mothers provided more childcare than fathers [53,54], other studies found an increase in father involvement in childcare at home [55,56].

### 4.4. Family Communication and Socialisation

Another finding of the current study suggested increased family communication patterns using technology and the lack of socialization between families during COVID-19. Lockdown restrictions have increased patterns of social distancing and self-isolation practices, to combat COVID-19. These restrictions and COVID-19 safety protocols further instigated new challenges for socialization and the formation of connections. Research evidence on the usage of technology, during COVID-19, emphasized the various roles of online social media platforms, to digitally re-connect with others. A study in the United States found that the social norms relative to communications and intimacy behavior have shifted in response to COVID-19 restrictions [57]. Watson et al. [57] further argued that social distancing behavior has prioritized the utilization of digital communication technologies, to maintain social connections. In support of these findings, a study in South Africa studied the changes in perceptions and the utilization of mobile technology and communication during COVID-19 restrictions. The study argues that lockdowns have constrained people to rely on digital technology for communication usage [58]. Although participants were knowledgeable on COVID-19, their use of web searches and social media outlets- as opposed to searching official government websites- can lead to misinformation of COVID-19 and related topics, being shared. Moor and March [59] similarly found social media to have a positive effect on building connections with loved ones and decreased feelings of isolation during COVID-19. This was especially found when using social media platforms to call offering strength and mental support to others. While technology largely facilitated increased online family routines and communication patterns, Kgatle [60] further underlined that South African churches have shifted to religious live-streaming, via social media platforms, to operate during South Africa’s COVID-19 lockdown. This, therefore, highlights that in South Africa, virtual forms of communication and digital technology use, have become the new norm for families. While increased usage of digital technologies has prioritized online forms of interactions, the current study found that COVID-19 social distancing and lockdown regulations, limited physical interactions with others. Loss of connections has been found to predispose individuals to factors of social isolation, loneliness, and depression [61]. David and Roberts [62] however argue that the more people socially distance themselves, the more socially disconnected they may feel. The study puts forth that the lack of social connection can trigger increased levels of stress and depression hindering people’s subjective well-being. Smartphones have therefore been viewed to moderate the relationship between social loss and social connection. However, David and Roberts [62] argue that although this alternative means to socially connect with people is not as effective as face-to-face interactions, it is helpful to utilize smartphones to socially connect during the COVID-19 pandemic.

### 4.5. Financial Management and Constraints

Contrasting to the findings above, another finding from the current study found that families have improved their financial management through learning how to save during lockdown regulation measures. In a study by Yuesti et al. [63], families improved and saved money through planning shopping trips, saving shopping receipts, and planning for the month or future ahead. This promoted financial literacy among family members and eventually helped families and individuals manage their income better, in demanding circumstances. Similarly, a study conducted in Indonesia based on families from various levels of socioeconomic status, found that financial behavior can greatly influence financial well-being [63]. For families in South Africa, employment status plays an essential role in family health and well-being which can largely promote family resilience [64]. However, educational attainment and unemployment rates are key concerns in South Africa [65]. Another key concern that was evident in the findings was the pandemic-linked factors of financial constraints. Globally, there have been increased rates of unemployment, in which global recession affected financial insecurity. This economic stress and adversity have been associated with poor health outcomes and parent-child relationships [66,67]. This in turn has affected marital conflicts and led to an increase in domestic violence [17]. In addition, a study review found various COVID-19 pandemic-associated socio-economic factors, that can impact families’ financial situations which can limit parent capacities to provide and promote caregiving, health, and well-being [68]. These impact factors of COVID-19 have further been associated with factors of job loss, food, and financial insecurity [69]. Poor financial resources have been found to make families vulnerable during the pandemic [70]. In addition, low-income families in the United States were found to experience job loss due to the pandemic and were unable to work from home [71]. These COVID-19 associated challenges have created greater financial hardships and stress amongst low-income families in the United States. Similarly, a study conducted in Soweto, South Africa found that increased perceived risk of COVID-19 infection was associated with an increase in depressive symptoms in adults specifically with traumatic childhood histories [72]. However, limited studies have been researched on the consequences of COVID-19 and mental health in sub-Saharan Africa [73].

### 4.6. Cleanliness and Good Health

Lastly, the current study found that families’ experiences of COVID-19 resulted in improved hygiene and health status at home. The study highlights the key role good hygiene and health status awareness played, in reducing poor health outcomes. In support of the current study’s findings, a study conducted in China found that hand sanitizing and wearing masks during COVID-19 was linked to decreased levels of stress, anxiety, and depression [74]. Similarly, Macaraan [75] argued that the increased awareness of the pandemic and behavioral changes in people’s personal hygiene and self-care habits have assisted in the prevention of avoiding diseases and the spreading of germs. Thus, underlining the importance of adjusted and adapted hygiene and health practices, in the promotion of better family and community health and wellness. However, contrasting to the good health outcomes associated with sanitizing and wearing masks, a study in Australia found that increased hygiene behaviors were linked to higher levels of anxiety and stress, underlining the poorer health outcomes, in relation to COVID-19 precautionary behaviors [76]. This denotes that enforced COVID-19 precautionary measures in hygienic behaviors may have different health outcomes, across diverse cultures.

## 5. Strengths and Limitations

The study of family life during COVID is an under-studied area of research. During this period, the family became especially important during periods of isolation and quarantine. This study of families is, therefore, a strength. In addition, qualitatively exploring the experiences of families, provides for a deeper understanding of the realities of COVID-19. Although COVID-19 is a global phenomenon, country-specific studies provide for the nuances of different experiences and add to the evidence of experiences around the world. Holistically, the current study adds to a growing body of science regarding, not only the family experience but also the research evidence about COVID-19.

Another strength of the study is that participants who are experiencing the phenomenon under study were interviewed whilst still experiencing the COVID pandemic while in the lockdown conditions. The experiences as relayed by the participants can therefore be interpreted as being close to their lived realities, as their recollections of it were still fresh in their minds. Also, participants were drawn from a large sampling frame. The large sampling frame strengthened the study because it allows the inclusion of a diverse population.

However, a limitation may be that due to COVID restrictions, the researchers had limited control over the recruitment of the participants, as they relied on the participants for referrals. The participants could have inadvertently referred potential persons or families whom they perceived to share a similar set of family dynamics or characteristics. Another limitation of the study was that interviews were conducted telephonically. This method of data collection prevented the interviewers from exploring all the non-verbal communications exhibited by the participants. Lastly, a follow-up study may be needed to assess the implications of COVID-19, once the pandemic has subsided, to truly identify the full implications of the pandemic on the lives of South African families.

## 6. Implications

Social conditions surrounding the COVID-19 pandemic have influenced family-related stressors and their overall well-being. The findings of the study further underline that family health plays an essential role in the wellbeing and functioning of families. However, the implications of COVID-19 have provided both good and challenging outcomes to the functioning of families and broader society. There is therefore a need for a greater exploration of the possible implications COVID-19 has on the diverse forms of families in South Africa. Future research could also employ a mixed-method approach to provide more complete evidence and an even richer understanding of the implications of this complex phenomenon, with its many unknowns. It should focus on whether these positive and negative effects, that were reported in the current study, persist even longer than the pandemic.

Interventions should be implemented to assist families to adapt to new daily routines forced upon them through lockdown, assist them in changing their methods of socialization and upholding and maintaining their relationships during the lockdown, as well as how to deal with family conflicts and financial constraints. Furthermore, implications for future research should include research on the health impacts of COVID-19 on diverse family structures, family compositions, and family dynamics. This study and other in-depth research can assist in developing policies and interventions for families.

## 7. Conclusions

COVID-19 and lockdown regulations have largely affected the economic, social, and health spheres in South Africa. The daily realities of South African families during COVID-19, have found that there are both beneficial and adverse factors experienced by families in relation to their socio-economic status, family connectedness, health, and well-being. Consequently, there remain concerns on the psychological impact of COVID-19 experienced by families. Although family time, family communication and adjustment, and the adoption of work environments and hygienic behavior, have facilitated and promoted positive health outcomes; policymakers should encourage interventions that promote family physical and mental health. The results highlight the need for more family-focused interventions and policies that focus on supporting families during COVID-19, to both address and reduce negative health outcomes experienced by families in South Africa.

## Figures and Tables

**Table 1:** Demographic characteristics of the participants.

Demographic Characteristic		Participants (*n* = 31)	Percentage (%)
Gender	Male	13	41.9
	Female	18	58.1
Age	18–24 years	4	12.9
	25–35 years	18	58.1
	36–46 years	3	9.7
	≥47 years	6	19.4
Highest educational level	High school	10	32.3
	Diploma/Certificate	5	16.1
	Bachelors/Honours	12	38.7
	Masters/Doctorate	4	12.9
Employment status	Employed	21	67.7
	Unemployed	10	32.3
Family structure	Nuclear family	22	71.0
	Single parent family	2	6.5
	Grandparent family	2	6.5
	Extended family	5	16.1
Home language	English	22	71
	Afrikaans	4	13
	Xhosa	3	10
	Zulu	1	3
	Other	1	3

**Table 2:** Interview schedule.

Examples of Questions
- What are the main concerns you and your family have with the COVID-19 pandemic? - What are the changes that you and your family have to make because of the COVID-19 pandemic? - How do you and your family experience these changes? - What are the positive aspects of the COVID-19 experience? - What are the negative aspects of the COVID-19 experience? - How do you and your family experience lockdown regulations?

**Table 3:** Summary of the study sub-themes.

Sub-Themes	Definition
Lockdown restriction on the family routines and events	This relates to routines such as going to work, school, and shop and events such as birthday celebrations and funerals
Family time	This refers to as time that families have to connect through various activities and events
Family communication and socialization	This denotes how and what families used for communication and socialization
Financial management and constraints	This denotes the financial challenges faced by families and how they were managed
Cleanliness and good health	This relates to personal and general hygiene
Psychological impact	This relates to the mental health and well-being of the families

## Data Availability

Upon reasonable request, transcripts of the study interviews are available from the corresponding author.
